# Diagnostic accuracy of diffuse reflectance imaging for early detection of pre-malignant and malignant changes in the oral cavity: a feasibility study

**DOI:** 10.1186/1471-2407-13-278

**Published:** 2013-06-05

**Authors:** Manju M Stephen, Jayaraj L Jayanthi, Nisha G Unni, Philip E Kolady, Valappil T Beena, Panniyammakal Jeemon, Narayanan Subhash

**Affiliations:** 1 Department of Oral and Maxillofacial Pathology, Government Dental College, Trivandrum, India; 2 Biophotonics Laboratory, Centre for Earth Science Studies, Akkulam, Trivandrum 695 011, India; 3 Institute of Cardiovascular and Medical Sciences, University of Glasgow, Glasgow, UK; 4 Centre for Chronic Disease Control, New Delhi, India; 5 Public Health Foundation of India, New Delhi, India; 6 Department of Surgical Oncology, Regional Cancer Centre, Trivandrum, India

**Keywords:** Sensitivity, Specificity, Diffuse reflectance imaging, Oral squamous cell carcinoma

## Abstract

**Background:**

Diffusely reflected light is influenced by cytologic and morphologic changes that take place during tissue transformation, such as, nuclear changes, extracellular matrix structure and composition as well as blood flow. Albeit with varying degree of sensitivity and specificity, the properties of diffusely reflected light in discriminating a variety of oral lesions have been demonstrated by our group in multiple studies using point monitoring systems. However, the point monitoring system could not identify the region with the most malignant potential in a single sitting.

**Methods:**

In order to scan the entire lesion, we developed a multi-spectral imaging camera system that records diffuse reflectance (DR) images of the oral lesion at 545 and 575 nm with white light illumination. The diagnostic accuracy of the system for 2-dimensional DR imaging of pre-malignant and malignant changes in the oral cavity was evaluated through a clinical study in 55 patients and 23 healthy volunteers. The DR imaging data were compared with gold standard tissue biopsy and histopathology results.

**Results:**

In total 106- normal/clinically healthy sites, 20- pre-malignant and 29- malignant (SCC) sites were compared. While the median pixel value of the R545/R575 image ratio for normal/clinically healthy tissue was 0.87 (IQR = 0.82-0.94), they were 1.35 (IQR = 1.13-1.67) and 2.44 (IQR = 1.78-3.80) for pre-malignant and malignant lesions, respectively. Area under the ROC curve to differentiate malignant from normal/clinically healthy [AUC = 0.99 (95% CI: 0.99-1.00)], pre-malignant from normal/clinically healthy [AUC = 0.94 (95% CI: 0.86-1.00)], malignant from pre-malignant [AUC = 0.84 (95% CI: 0.73-0.95)] and pre-malignant and malignant from normal/clinically healthy [AUC = 0.97 (95% CI: 0.94-1.00)] lesions were desirable.

**Conclusion:**

We find DR imaging to be very effective as a screening tool in locating the potentially malignant areas of oral lesions with relatively good diagnostic accuracy while comparing it to the gold standard histopathology.

## Background

Oro-pharyngeal cancer is a major component of the global cancer burden [1,2]. Early detection of pre-malignant changes facilitates timely adoption of preventive measures and treatment strategies [3]. The potential beneficial effect of early detection and treatment may improve the survival rates in patients with cancer of the oral cavity [4]. The gold standard for diagnosis of oral cavity cancer is an invasive tissue biopsy and histopathology examination. However, detection of the potentially malignant site for biopsy is visually challenging even for experienced clinicians, which often leads to multiple biopsies and delay in diagnosis.

Toluidine blue staining [5] and direct fluorescence visualization [6,7] have been used in clinical settings as an adjunctive visual tool to enhance the contrast between the clinical lesions and the adjacent normal oral tissue. Diffusely reflected (DR) white light spectra were also studied by various groups [8,9] for tissue differentiation in oral cavity. Several multi-centric clinical studies established the effectiveness of optical spectroscopy techniques for non-invasive detection of oral malignancies with good diagnostic accuracies [10-14]. However, they are point monitoring systems that analyse the tissue characteristics at a particular point in the entire area of an oral lesion.

Several spectroscopic imaging techniques that rely on tissue fluorescence have emerged recently for the detection of oral malignancies [6,15-17]. Our previous experience suggests that the diagnostic accuracies based on DR spectroscopy are superior to fluorescence spectroscopy in point monitoring systems. We therefore developed a multi-spectral diffuse reflectance imaging system for recording of DR images in vivo and report the diagnostic accuracies of this system in comparison to the gold standard tissue biopsy and histopathology examination.

## Methods

### Diffuse reflectance imaging system

**Figure 1:**
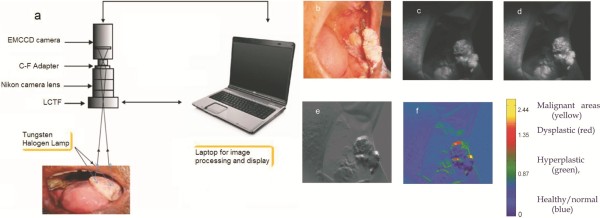
**Diffuse reflectance (DR) imaging set-up and image processing.** (**a**) Schematic of the diffuse reflectance imaging system (DRIS) and (**b**) photo of the oral cavity of a patient with verrucous growth in the left commissure, typical set of monochrome images recorded (**c**) at 545 and (**d**) 575 nm, (**e**) computed DR ratio (R545/R575) image and (**f**) pseudo colour mapped ratio image. Color palette shows the range of median pixel intensity values for lesion discrimination.

The diffuse reflectance imaging system (DRIS) [18] developed (Figure 1) for recording the monochrome images of the oral cavity lesion at 545 and 575 nm in the present study consisted of an electron multiplying charge coupled device (EMCCD) camera (model: LUCA-R, Andor Technology, UK) with 1024 × 1024 pixels of 8 micron size, coupled to a Nikkon AF 35–70 zoom lens for focusing and a liquid crystal tunable filter (LCTF) of 7 nm bandwidth (CRI Inc., USA) for wavelength selection. Suitable adapters were used to connect the camera with the focusing lens and the LCTF, and roller ball assemblies were built to facilitate camera movement during focusing/zooming. The tungsten halogen lamp (12 V, 55 W) that comes as standard white light in dental chairs was used for illumination of oral cavity during imaging. A laptop computer working with the SOLIS program (Andor Technology, UK) controlled the image acquisition parameters, recorded the images sequentially at 545 and 575 nm and computed the ratio image (R545/R575) arithmetically. The spatial distribution of the image ratio R545/R575 of the lesion was displayed as a Pseudo Color Map (PCM) according to the ratio value of each pixel in the image and based on cut-off values derived from our previous studies [11-14] with a point monitoring system. Thus, PCM classified the oral lesion into blue (healthy tissue), red (dysplastic/ pre-malignant) and yellow (malignant tissue) colors, thereby providing a visual discerning capacity to the eye in differentiating oral lesions [19] and identifying regions with maximum potential to show dysplastic characteristics and invasion.

### Study settings and study measurements

The present study was conducted at the Oral and Maxillofacial Pathology Department of the Government Dental College (GDC), Trivandrum, after obtaining the Ethical clearance from the GDC Ethics committee (No. IEC/C/28-A/2010/DCT), and strictly adhering to the approved protocols. Consecutive patients were invited to participate in the study. Before enrollment, the study subjects were asked to rinse their mouth with 0.9% saline solution. A clinician then examined the oral cavity of the patient for suspicious lesions, including white/red patches, mixed white and red lesions, non-healing ulcers and mucosal growth. The study procedures were explained to the patient and written informed consent was obtained from all participants before initiating any study related measurements. All patients enrolled were more than 20 years of age, and had no previous history of cancer or radiation/ chemotherapy treatment, or used any medication orally for the past seven days, and had no life threatening medical condition. Patient details were recorded and the clinical characteristics of the lesions were noted by a qualified clinician. There was no refusal to participate in the study. The DR imaging system was used to capture monochrome images of all suspected oral lesions at 545 and 575 nm. Tissue biopsies were taken from the region with maximum potential to show dysplastic characteristics in a lesion as identified by the PCM of the DR image ratio R545/R575. DR images recorded from 13 different anatomical sites of healthy volunteers were used as site-specific controls. Tissue biopsy was not taken for healthy volunteers and visual examination report of the pathologist was used for categorization of the tissue as clinically healthy.

The biopsy samples were fixed in 10% formalin and sent to the laboratory for pathological analysis. The tissue samples were then dehydrated in ascending grades of alcohol, embedded in paraffin block, micro-sectioned using microtome and mounted on a thin glass plate. This was then stained using haematoxylin and eosin stains. Two oral pathologists, who were blinded to the spectral measurement details, prepared the slides and provided the histo-pathological results independently. A third opinion was sought if there was any disagreement between the two independent reports.

The DR image ratio was further analyzed based on the mean pixel intensity of 100 pixels at 30 different points within the proposed biopsy site. Median value of the image ratio from these 30 points were determined and used for classifying the lesions into three groups, viz. normal/clinically healthy, pre-malignant or malignant (squamous cell carcinoma: SCC). Oral lesions were classified according to the image ratio value R545/R575 and the results were correlated with histo-pathologic findings to determine the diagnostic accuracies.

### Statistical analyses

The characteristics of the study population are summarized as means and percentages. The DR spectral data were compared across different tissue types based on the histo-pathology findings using scatter plot. Receiver Operator Characteristics (ROC) curves were also constructed using the DR spectral data to differentiate pre-malignant from normal/clinically healthy tissue. The area under the ROC curves (AUC) and its 95% CI were computed. The statistical significance of AUC was tested using non-parametric assumptions.

## Results

All together 55 patients with oral lesions, such as erythroplakia, leukoplakia, non-healing ulcers, and mucosal growth, and 23 healthy volunteers as control were enrolled for the study. The study was carried out from April 2010 to December 2010. While the controls were significantly younger than cases, tobacco use and alcohol use were reported more frequently in cases than controls (Table 1). Out of the 55 patients examined using DR imaging, the pathology report showed that 6 lesions were epithelial hyperplasias with no dysplasia, 20 lesions were epithelial hyperplasia with different grades of dysplasia (mild, moderate and severe), and 29 lesions were malignant, which included well differentiated, moderately differentiated and poorly differentiated SCCs (Characteristics of the lesions are described in supplementary Additional file 1: Table S1). Epithelial hyperplasia with no dysplasia were included in the group of healthy and designated as normal/clinically healthy for analysis purpose. A total of 100 sites were examined from 23 healthy volunteers. Thus, the analysis was finally conducted by comparing 106- normal/clinically healthy sites, 20- pre-malignant (dysplasia) and 29- malignant (SCC) sites.

**Table 1:** Characteristics of study population categorized according to the grade of malignancy

**Patient characteristics**	**Normal/clinically healthy**	**Pre-malignant**	**Malignant (cancer)**
Age (mean, SD)	27.5 (7.0)	54.2 (12.0)	57.2 (10.5)
Men (n, %)	17 (16.5)	13 (59.1)	14 (42.4)
Tobacco use (n, %)	1(1)	17 (77.3)	26 (78.8)
Alcohol use (n, %)	13 (12.6)	8 (36.4)	15 (45.5)

**Figure 2:**
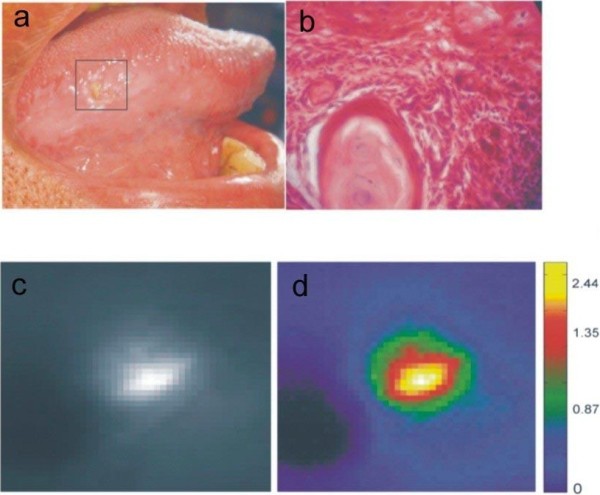
Imaging results of a typical malignant tongue lesion; (a) photograph of the traumatic ulcer on the right lateral border of the tongue, (b) pathological investigation shows as well-differentiated SCC (WDSCC), (c) monochrome ratio image R545/R575 and (d) PCM ratio image shows the most malignant area in light yellow.

A non-healing ulcerated lesion in a 63 year old male patient on the right lateral border of the tongue that was present for more than two months is presented in Figure 2 along with its monochrome and false colored image ratios. While the bright yellow spot in the PCM indicates malignancy, the histo-pathological analysis of the biopsy sample confirms the diagnosis of well differentiated SCC (WDSCC).

**Figure 3:**
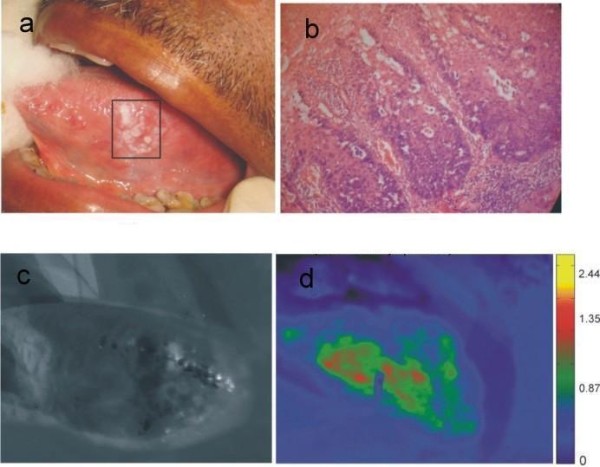
Imaging results of a typical pre-malignant lesion; (a) photograph of the lesion (leukoplakia on the LT of a 43 old male), (b) pathology shows severe dysplasia, (c) monochrome ratio image R545/R575 and (d) PCM ratio image (R545/R575) shows severe dysplastic region as red.

By contrast, a lesion on the left lateral surface of the tongue in a 43 year old male patient, clinically diagnosed as speckled leukoplakia, histo-pathologically exhibits severe dysplastic features (Figure 3). The PCM image of this lesion shows red areas categorizing the lesion as pre-malignant.

**Figure 4:**
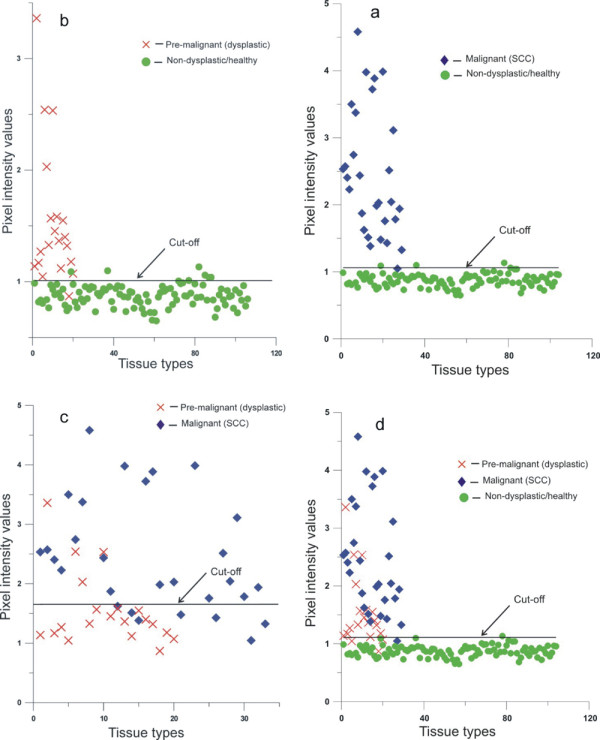
**Scatter plot diagram of pixel intensity values of the ratio image; (a) malignant and normal/clinically healthy, (b) pre-malignant and normal/clinically healthy, (c) pre-malignant and malignant and (d) malignant or pre-malignant and normal/clinically healthy.** The cut-off lines for discrimination are drawn at the mean ratio value of the groups classified.

**Figure 5:**
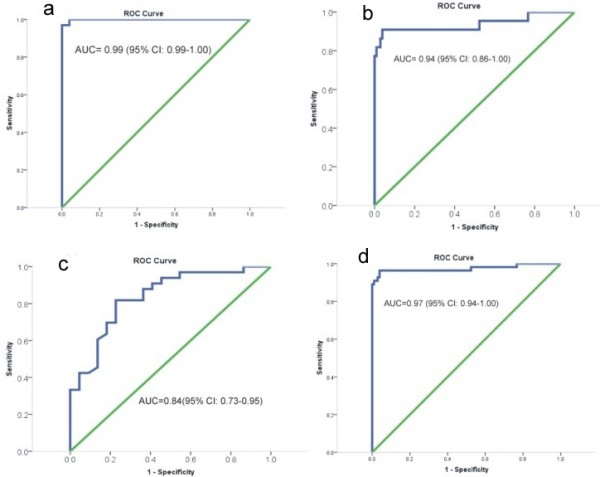
ROC curves showing the discriminatory capacity of the properties of ratio-image to differentiate; (a) malignant lesions from normal/clinically healthy tissue, (b) pre-malignant lesions from normal/clinically healthy tissue, (c) pre-malignant lesions from malignant and (d) malignant and pre-malignant from normal/clinically healthy tissue.

While the median pixel value of the R545/R575 image ratio for normal/clinically healthy tissue was 0.87 (IQR = 0.82-0.94), they were 1.35 (IQR = 1.13-1.67) and 2.44 (IQR = 1.78-3.80) for pre-malignant and malignant lesion, respectively. Scatter plots based on R545/R575 image ratio are presented in Figure 4. While 1/29 malignant cases was misclassified as normal/clinically healthy, 3/106 normal/clinically healthy lesions were misclassified as malignant at a mean pixel intensity cut-off of 1.1 (97% sensitivity and specificity). Similarly a cut-off of 1.01 (Figure 4b) miss-classified 1/20 pre-malignant cases as normal/clinically healthy and 8/106 normal/clinically healthy cases as pre-malignant (sensitivity of 95% and specificity of 92%). While a cut-off at 1.66 (Figure 4c) misclassified 7/29 malignant lesions as pre-malignant and 4/20 pre-malignant lesions as malignant (sensitivity of 76% and specificity of 80%), a cut-off at 1.06 (Figure 4d) misclassified 4/49 cancerous or pre-malignant cases as normal/clinically healthy and 5/106 normal/clinically healthy cases as cancerous or pre-malignant (sensitivity of 92% and specificity of 95%). Discordant findings of histopathology and DR imaging results are presented in Table 2 with their detailed histopathology reports. Diagnostic accuracies and positive and negative predictive values are presented in Table 3. Area under the ROC curves (Figure 5a-d) show the discriminatory capacity of the image ratio to differentiate malignant from normal/clinically healthy [AUC = 0.99 (95% CI: 0.99-1.00)], pre-malignant from normal/clinically healthy [AUC = 0.94 (95% CI: 0.86-1.00)], malignant from pre-malignant [AUC = 0.84 (95% CI: 0.73-0.95)] and pre-malignant and malignant from normal/clinically healthy [AUC = 0.97 (95% CI: 0.94-1.00)] lesions.

**Table 2:** Discordant findings of histopathology and DR imaging results

**Case no**	**Clinical diagnosis**	**Histopathological (microscopic examination) report**	**DR image ratio result**
**5**	Proliferative growth	Tumor epithelial cells were seen invading into the underlying connective tissue in the form of cords, islands, strands and nests. The invading cells exhibited features of pleomorphism, hyperchromasia, increased nuclear to cytoplasmic ratio and abnormal mitotic figures. Few keratin pearl formations were evident. Features suggestive of MDSCC.	Normal
12	Verrucous growth	Breach in the basement membrane was evident and tumor epithelial cells were seen invading into the underlying connective tissue in the form of islands, strands and nests. Keratin pearl formations were few. Tumor epithelial cells showed hyperchromasia, increased nuclear to cytoplasmic ratio, pleomorphism and abnormal mitotic figures. Features suggestive of MDSCC.	Normal
18	Non-specific ulcer	The epithelium was hyperchromatic in the basilar one-third region. Dense chronic inflammatory cells were seen within the connective tissue in the ulcerated region. Features suggestive of epithelial hyperplasia with mild dysplasia.	Normal
23	Non-healing ulcer	Epithelium exhibits no features of dysplasia. Moderate collections of chronic inflammatory cells were seen in the juxta epithelial region. Features suggestive of EHP with no dysplasia.	Pre-malignant
36	Ulcerated lesion with keratotic border	Epithelium exhibits features of hyperchromasia, increased mitotic figures, increased nuclear; cytoplasmic ratio, increased basilar hyperplasia involving two third of the epithelium. Chronic inflammatory cells were moderately dispersed in the juxta epithelial region. Features suggestive of EHP with moderate dysplasia.	Normal
39	Non-specific ulcer	Basal and suprabasal cells exhibited hyperplasia and hyperchromasia. The inflammatory cells were chronic and diffusely spread within the connective tissue. Features suggestive of EHP with mild dysplasia.	Normal

MDSCC=moderately differentiated squamous cell carcinoma and EHP=epithelial hyperplasia.

**Table 3:** Diagnostic accuracies of DRIS in discriminating lesions

**Lesion types**	**Diagnostic accuracies**				**Area under the ROC curve**
	**Sensitivity (%)**	**Specificity (%)**	**PPV (%)**	**NPV (%)**	
Malignant Vs Normal	97	97	90	99	0.99
Pre-malignant Vs Normal	95	92	70	99	0.94
Pre-malignant Vs malignant	76	80	85	70	0.84
Malignant & Pre-malignant Vs Normal	92	95	90	96	0.97

PPV=positive predictive value, NPV=negative predictive value and ROC=receiver operator characteristics.

## Discussion

The present study examines the diagnostic accuracy of a spectral imaging system based on the principles of diffuse reflectance in discriminating healthy oral tissue from pre-malignant and malignant tissues. The diagnostic accuracy obtained in this study is superior to other imaging systems for detection of malignant changes in the oral cavity.

When light interacts with biological tissue a small portion of it is absorbed or transmitted while the rest undergoes multiple elastic scattering due to heterogeneity in the refractive index of the tissue components and gets diffusely reflected. Often, a portion of the impinging radiation is reflected from the surface when the roughness of the boundary is small in comparison with the wavelength of light. The portion that penetrates the sample gets scattered at a larger number of points in its path due to uneven, broken or bumpy boundary surfaces, where the coarseness is of the same order of magnitude as the wavelength. During tissue transformation healthy tissue undergoes morphometric and cytologic changes such as increase in epithelial thickness, nuclear size, nuclear to cytoplasmic ratio, changes in the chromatin texture and collagen content, and angiogenesis [20-22]. These changes modify the diffusely reflected component of the incoming radiation. We hypothesized that the diffuse reflectance intensity decreases during the transformation of tissue from healthy to dysplastic due to morphologic, cytologic and vascular changes associated with transformation. While our previous studies supported this hypothesis and differentiate pre-malignant and malignant tissue with relatively good sensitivity and specificity, the present study findings further strengthens this relationship and enables identification of areas with the most malignant potential in an oral lesion.

**Figure 6:**
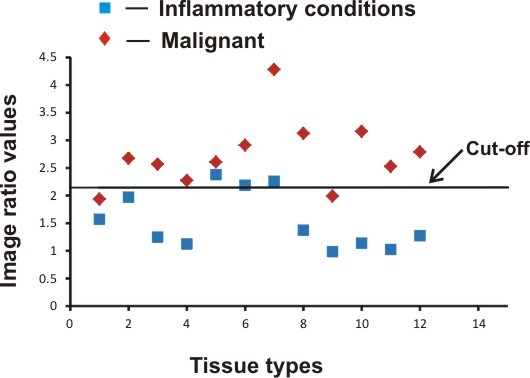
Scatter plot diagram of pixel intensity values for the image ratio (R545/R575) discriminating malignant and inflammatory areas.

Although the biological mechanisms associated with changes in DR of different tissue types are not clear, one potential possibility is associated with changes in production of haemoglobin. In malignant tissues, the haeme synthesis is disturbed due to the reduced activity of the ferrochelatase enzyme [23] that results in lower haemoglobin production and correspondingly lower absorption at 545 and 575 nm of the oxygenated haemoglobin spectra. A reduction in oxygenated haemoglobin increases the DR ratio of R545/R575. Conversely, during inflammatory conditions there is an increase in haeme production, which leads to an enhancement in the oxygenated haemoglobin and concomitant decrease in the DR ratio of R545/R575 (Figure 6).

The diagnostic accuracies obtained in our study are superior to the diagnostic accuracies obtained in studies using direct tissue fluorescence visualization [6,16,17]. Unlike the present study, the discriminative capacity was tested in a homogenous group of malignant lesions consisting of mainly severe dysplastic tissues in all other studies. The high diagnostic accuracy obtained in our study underlines the potential use of this method in routine clinical practice. However, we could not perform site-specific analyses due to small number of cases in each anatomical location. The diagnostic accuracies presented in our study are therefore averaged for all types of lesions of the oral cavity and it would have been better if site-specific lesion classifications were possible.

The basis for our DR imaging method arises from the differences in the spectra obtained from the normal and diseased tissue due to the multiple physiological changes associated with tissue transformation from healthy to pre-malignant/dysplastic and malignant. The imaging method has the advantage of non-invasively scanning the entire lesion and its surrounding areas in real-time, and categorize oral lesions into normal/clinically healthy, pre-malignant and malignant tissue. Furthermore, it efficiently delineates the boundaries of neoplastic changes and locates the site with most malignant potential for a biopsy, thereby avoiding unnecessary repeated biopsies and delay in diagnosis. Imaging the entire region may also help the surgeons to identify the margins of the lesion that cannot be easily visualized by the naked eye during surgical interventions. Applications of digital image processing techniques may further enhance our ability to objectively identify and delineate the peripheral extent of neoplastic lesions.

Since clinical diagnosis based on DR imaging is possible in near real-time, there is practically no waiting period for the patient. The method is relatively cheap and can be implemented in all clinical settings with minimal training. Training the non-physician health workers in the imaging technique and screening the patients for tissue biopsy may further reduce the cost. However, the cost-effectiveness of mass screening of oral lesions using the imaging technique needs to be evaluated in different settings before its wider adaptation. Furthermore, the multi-spectral DR imaging technique presented in this paper has the potential to be used as an adjunct to colposcopy in the screening of cervical pre-cancers and in the identification the most malignant site for biopsy. Further studies in a larger population may help us to develop better classification algorithms for discriminating dysplastic lesions as mild, moderate and severe, and SCC lesions as ‘well differentiated’, ‘moderately differentiated’ and ‘poorly differentiated’ tissue categories.

## Conclusion

Diffuse reflectance spectral imaging technique efficiently differentiates healthy tissue from pre-malignant and malignant tissue in the oral cavity. The imaging system developed for this study can be used as an adjunctive visual tool to enhance the contrast between dysplastic tissue and normal tissue in oral cavity lesions in clinical settings.

## Competing interests

P Jeemon is supported by Wellcome Trust capacity building strategic award to the Public Health Foundation of India. Subhash N., and Jayanthi J.L., have applied for patent for the diffuse reflectance imaging system (DRIS) developed for recording the monochrome images of the oral cavity lesion at 545 and 575 nm in the present study (Indian Patent Appl. No. 2870/CHE/2011, Filed on 23.08.2012 at the Patent Office, Chennai).

## Authors’ contributions

NS, MS and VTB conceived the experimental concept. MS, JLJ and NGU set up the experiment and acquired experimental spectral data. Tissue biopsy and histo-pathology were performed by MS and PEK. Data were analysed and interpreted by PJ, JLJ, MS, VTB, PEK and NS. All authors had full access to the data and contributed to the manuscript writing and revisions. The final manuscript was reviewed and approved by all authors. NS is the guarantor of the study.

## Pre-publication history

The pre-publication history for this paper can be accessed here:

http://www.biomedcentral.com/1471-2407/13/278/prepub

## Supplementary Material

Additional file 1: Table S1 Patient details with their clinical, pathological and study results. Click here for file
