# Supplementary material for: Diagnostic accuracy of diffuse reflectance imaging for early detection of pre-malignant and malignant changes in the oral cavity: a feasibility study
Source: BMC Cancer. 2013 Jun 5;13:278. doi: 10.1186/1471-2407-13-278 (PMC3679752; doi:10.1186/1471-2407-13-278)
Supplement: Additional file 1: Table S1 — Patient details with their clinical, pathological and study results. [file 1471-2407-13-278-S1.doc]

| **Table S1.** Patient details with their clinical, pathological and study results | | | | |
| --- | --- | --- | --- | --- |
| Patient No | Clinical diagnosis | Histopathological reports | Image ratio value | Study Result |
| 1 | Ulcero-proliferative lesion | MDSCC | 2.40468 | SCC |
| 2 | Leukoplakia | EHP with moderate dysplasia | 1.13358 | Dysplasia |
| 3 | Carcinoma | MDSCC | 2.24485 | SCC |
| 4 | Proliferative growth | MDSCC | 2.74446 | SCC |
| 5 | Proliferative growth | MDSCC | 0.88194 | Hyperplasia |
| 6 | Leukoplakia | EHP with mild dysplasia | 1.26902 | Dysplasia |
| 7 | Leukoplakia | WDSCC | 7.03295 | SCC |
| 8 | Traumatic ulcer | WDSCC | 2.43627 | SCC |
| 9 | Traumatic ulcer | WDSCC | 4.00488 | SCC |
| 10 | Leukoplakia | EHP with moderate dysplasia | 2.52826 | Dysplasia |
| 11 | Leukoplakia with OSMF | EHP with mild dysplasia | 2.01109 | Dysplasia |
| 12 | Verrucous growth | MDSCC | 0.80603 | Hyperplasia |
| 13 | Leukoplakia | EHP with mild dysplasia | 1.32833 | Dysplasia |
| 14 | Carcinoma | MDSCC | 2.7742 | SCC |
| 15 | Ulcerated lesion with keratotic border | MDSCC | 2.2732 | SCC |
| 16 | Leukoplakia | EHP with mild dysplasia | 1.37276 | Dysplasia |
| 17 | Ulcerated lesion with keratotic border | MDSCC | 1.70434 | SCC |
| 18 | Non-specific ulcer | EHP with mild dysplasia | 0.93364 | Dyperplasia |
| 19 | Non-healing ulcer | WDSCC | 1.42418 | SCC |
| 20 | Leukoplakia | WDSCC | 2.53252 | SCC |
| 21 | Leukoplakia | EHP with mild dysplasia | 1.37668 | Dysplasia |
| 22 | Erythroluekoplakia | PDSCC | 2.04958 | SCC |
| 23 | Non-healing ulcer | EHP with no dysplasia | 1.58292 | Dysplasia |
| 24 | Leukoplakia | WDSCC | 1.13813 | SCC |
| 25 | Carcinoma | MDSCC | 3.05228 | SCC |
| 26 | Leucoplakia | EHP with mild dysplasia | 1.18332 | Dysplasia |
| 27 | Carcinoma | MDSCC | 1.33544 | SCC |
| 28 | Speckled leukoplakia | PDSCC | 3.78023 | SCC |
| 29 | Erythroplakia | EHP with severe dysplasia/CIS | 1.58312 | Dysplasia |
| 30 | Ulcero-proliferative growth | EHP with no dysplasia | 0.87621 | Hyperplasia |
| 31 | Ulcerated lesion | EHP with moderate dysplasia | 1.43691 | Dysplasia |
| 32 | Leukoplakia | EHP with no dysplasia | 0.85342 | Hyperplasia |
| 33 | Carcinoma | WDSCC | 2.06783 | SCC |
| 34 | Verrucous leukoplakia | MDSCC | 2.86751 | SCC |
| 35 | Verrucous growth | PDSCC | 3.78005 | SCC |
| 36 | Ulcerated lesion with keratotic border | EHP with moderate dysplasia | 0.87540 | Hyperplasia |
| 37 | Leukoplakia with erosive area | WDSCC | 1.89052 | SCC |
| 38 | Carcinoma | MDSCC | 2.57845 | SCC |
| 39 | Non-specific ulcer | EHP with mild dysplasia | 0.82310 | hyperplasia |
| 40 | Ulcero-proliferative growth | WDSCC | 1.98076 | SCC |
| 41 | Ulcerated lesion | EHP with moderate dysplasia | 1.49364 | Dysplasia |
| 42 | Carcinoma | MDSCC | 2.76510 | SCC |
| 43 | Speckled leukoplakia | EHP with severe dysplasia/CIS | 1.60431 | Dysplasia |
| 44 | Verrucous leukoplakia | WDSCC | 2.06251 | SCC |
| 45 | Leukoplakia with keratotic border | EHP with no dysplasia | 0.845672 | Hyperplasia |
| 46 | Traumatic ulcer | EHP with mild dysplasia | 1.15570 | Dysplasia |
| 47 | Erythroleukoplakia | EHP with moderate dysplasia | 1.48620 | Dysplasia |
| 48 | Ulcero-proliferative growth | WDSCC | 2.13421 | SCC |
| 49 | Ulcerated lesion | EHP with moderate dysplasia | 1.48202 | Dysplasia |
| 50 | Verrucous carcinoma | MDSCC | 2.76430 | SCC |
| 51 | Non-healing ulcer | EHP with severe dysplasia/CIS | 1.50327 | Dysplasia |
| 52 | Leukoplakia | EHP with no dysplasia | 0.86523 | Hyperplasia |
| 53 | Ulcero-proliferative growth | WDSCC | 1.96731 | SCC |
| 54 | Leukoplakia with erosive area | EHP with moderate dysplasia | 1.47612 | Dysplasia |
| 55 | Non-healing ulcer | EHP with no dysplasia | 0.90452 | Hyperplasia |
| CIS-Carcinoma in situ, EHP-Epithelial hyperplasia, SCC-Squamous cell carcinoma, WDSCC-Well differentiated SCC, MDSCC-Moderately differentiated SCC, PDSCC-Poorly differentiated SCC | | | | |
